# ATP-Independent Initiation during Cap-Independent Translation of m^6^A-Modified mRNA

**DOI:** 10.3390/ijms22073662

**Published:** 2021-04-01

**Authors:** Pavel A. Sakharov, Egor A. Smolin, Dmitry N. Lyabin, Sultan C. Agalarov

**Affiliations:** Institute of Protein Research, Russian Academy of Sciences, 4 Institutskaya St., 142290 Pushchino, Russia; p_sakharov@inbox.ru (P.A.S.); smolinegoralexeyevich@gmail.com (E.A.S.); sultan@vega.protres.ru (S.C.A.)

**Keywords:** 48S complex assembly, translation initiation, m^6^A-modified RNA, toeprinting

## Abstract

The methylation of adenosine in the N^6^ position (m^6^A) is a widely used modification of eukaryotic mRNAs. Its importance for the regulation of mRNA translation was put forward recently, essentially due to the ability of methylated mRNA to be translated in conditions of inhibited cap-dependent translation initiation, e.g., under stress. However, the peculiarities of translation initiation on m^6^A-modified mRNAs are not fully known. In this study, we used toeprinting and translation in a cell-free system to confirm that m^6^A-modified mRNAs can be translated in conditions of suppressed cap-dependent translation. We show for the first time that m^6^A-modified mRNAs display not only decreased elongation, but also a lower efficiency of translation initiation. Additionally, we report relative resistance of m^6^A-mRNA translation initiation in the absence of ATP and inhibited eIF4A activity. Our novel findings indicate that the scanning of m^6^A-modified leader sequences is performed by a noncanonical mechanism.

## 1. Introduction

Although the methylation of adenosine in the N^6^-position (m^6^A) modification was originally identified back in the 1970s [1], its abundance and role in the regulation of gene expression have been revealed recently [2,3], along with the finding that m^6^A enriches 5′- and 3′-untranslated regions (UTRs) of mRNA and stop-codon-adjacent sequences [2,3,4,5,6]. In mRNA, the methylation of adenosine in the N^6^ position results from the enzymatic activity of methyltransferases and demethylases. In mammalian mRNA, a methyl group appearance in the N^6^ position of adenosine is catalyzed by the heterodimers METTL3 and METTL14, the latter being associated with a pool of proteins where WTAP is crucial [7,8]. The cessation of methylation can be caused by the demethylases FTO or ALKBH5 (both from the ALKB family) [9,10]. The m^6^A is recognized by many proteins that frequently mediate its function. Specifically, YTHDF1 supports the translation of m^6^A-modified mRNAs by recruiting the initiation factor eIF3 into the 5′ UTR, thus involving the 43S pre-initiation complex [11]. The m^6^A modification also affects the mRNA stability as the average half-life of m^6^A-modified transcripts is shorter than nonmodified ones. The degradation of these transcripts is mediated by the protein YTHDF2, which preferably binds to m^6^A-containing transcripts and moves them into P-bodies or stress granules [12]. YTHDF2 can recruit the deadenylase complex CCR4–NOT through direct interaction with CNOT1, thereby apparently initiating deadenylation and mRNA degradation [13,14].

The m^6^A modification affects, directly or indirectly, the regulation of mRNA translation and stability and many other cellular events, including splicing and RNA export [11,12,15,16,17,18]. By itself, this modification does not affect the translation accuracy, probably because a significant proportion of methylation occurs in untranslated regions. However, the additional methyl group changes the AU-pair energetics and destabilizes uracil pairing [19]. Therefore, the presence of m^6^A in mRNA can be structure-affecting, thus altering the accessibility of motifs to RNA-binding proteins. For example, the global decrease in the m^6^A level masks a considerable subgroup of hnRNP C binding sites, thereby affecting the alternative splicing [20].

Mapping of m^6^A in the transcriptome identified a subset of m^6^A sites located in the mRNA 5′ UTR [2,3] that is crucial for ribosome recruiting. As a rule, translation begins when the 43S preinitiation complex is recruited into mRNA by the complex of translation initiation factors eIF4F (eIF4E + eIF4G + eIF4A) associated with the cap structure and the adjacent mRNA region. Under stress, translation can be independent of the cap structure and the cap-binding initiation factor eIF4E [21,22,23].

m^6^A modification has lately been considered responsible for switching from the cap-dependent mechanism of translation initiation to a cap-independent one, as well as translation of particular mRNAs in stress conditions. It was shown that the presence of m^6^A in the mRNA 5′ UTR ensures the direct eIF3-to-mRNA binding, which proves to be sufficient for translation initiation through the recruitment of the 43S preinitiation complex [24]. It was also found that eIF3 preferably binds mRNA transcripts that have m^6^A in the 5′ UTR. The effect of the 5′ UTR m^6^A on translation initiation was also reported by Coots et al. [25]. They showed that in the case of disturbed eIF4F-dependent translation initiation, cells use another mechanism, which is neither cap- nor IRES (internal ribosome entry site)-dependent, employing ^6^A-methylation of mRNA. This study identified the protein ABCF1 as the crucial mediator of the initiation of m^6^A–RNA translation that serves as an alternative recruiter of the ternary complex (TC) during the noncanonical initiation of translation. So far, the literature offers only one study on the mechanism of initiation of m^6^A–mRNA translation. Using both in vitro reconstitution approaches and translation assays, Meyer et al. [24] showed that methylated *Fluc* mRNA carrying the 5′ UTR from *beta-globin* mRNA is translated by a cap-independent mechanism; the translation occurs by a 5′-end-dependent mechanism that probably involves scanning of the reporter mRNA 5′ UTR, which apparently may happen without the participation of eIF4F. However, the role of the initiation factor eIF4A, and whether ATP hydrolysis was required for scanning remained obscure. Additionally, experiments on the translation of methylated mRNA used a rather unusual translation system based on cellular lysates deficient in eIF4E activity.

Our study confirms that ^6^A-methylated mRNA can be translated despite the suppressed mechanism of cap-dependent translation, although the efficiency of translation initiation is lower than that of unmodified mRNA in standard conditions. The major novel finding of our work is that for methylated leader sequences, scanning does not necessarily recruit ATP.

## 2. Results

### 2.1. The Toeprinting Assay of Initiation on m^6^A-Modified mRNA

**Figure 1:**
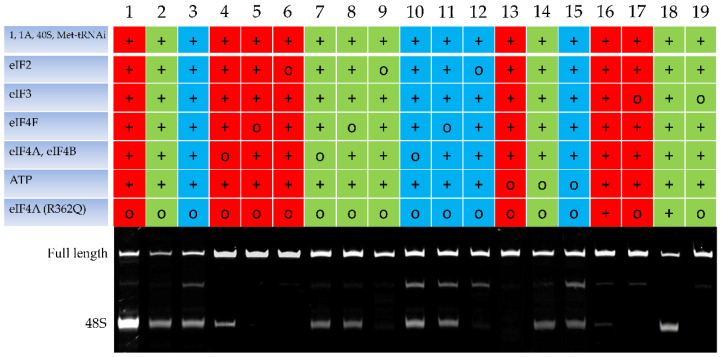
The formation of 48S complexes on capped mRNA with the rabbit beta-globin leader sequence. Toeprinting electropherograms (denaturing 6% PAGE, GelDoc Systems (BioRad, USA) visualization) are presented. Toeprints were detected by fluorescence; the fluorescent dye (FAM) was within the reverse transcription primer. Full-length cDNA and the 48S complex are indicated. The addition of components is denoted as +, their absence as 0. Red lanes, capped unmodified mRNA; green lanes, capped m^6^A-modified mRNA; azure lanes, uncapped m^6^A-modified mRNA. Detailed examination of toeprinting electropherograms (lanes 3, 10, 11, 12, and 15) revealed additional bands between the 5′ and 3′ ends of modified mRNA. These stops in reverse transcription were observed previously and associated with the presence of a stretch of adjacent modified nucleotides m^6^A(4) [24], which were poorly recognized by the reverse transcriptase and caused the termination of reverse-transcription elongation.

To reveal the peculiarities of m^6^A-modified mRNA translation initiation, we used an in vitro system based on individual initiation factors from rabbit reticulocyte lysates [26]. We experimented on mRNA containing the rabbit beta-globin leader sequence and a part of the beta-globin coding sequence. Three variants of this mRNA were used: one was a capped modification-free control, and two others were capped and uncapped forms with ^6^A methylation. After incubation in the reaction mixture to form 48S complexes, the toeprinting assay was performed using a fluorescein-labeled primer [27]. The toeprints were visualized using denaturing electrophoresis, followed by the detection of cDNA by fluorescence. Capillary electrophoresis with fluorescence detection was used to quantify the ratio of toeprinting products [28]. As follows from the toeprinting electropherogram (Figure 1, tracks 1–3) with the complete set of the initiation factors present, the 48S complex was formed on all the used mRNAs, as evidenced by the two bands corresponding to the full-length reverse-transcription product (upper band) and a truncated cDNA fragment (lower band), that is, the 48S complex. The specificity of the formed 48S complexes was confirmed experimentally using an eIF2-deficient system. As shown in Figure 1 (tracks 6, 9, 12) that in the absence of eIF2, no truncated toeprinting product was formed on any of these mRNAs; there is only one band corresponding to the full-length reverse transcription product. The experiments on the unmodified mRNA (Figure 1, track 5) reproduced our previous results [29] showing that the initiation of the translation of mRNA with the beta-globin leader sequence is cap-dependent and eIF4F-dependent (the removal of eIF4F results in the disappearance of the 48S-corresponding band). Additionally, for this mRNA, we identified the ATP dependence of the translation initiation (Figure 1, track 13) since no 48S complex was formed in the absence of ATP. The addition of mutant eIF4A (R362Q), an inhibitor of the ATP-dependent scanning (Figure 1, track 16), entailed the initiation inhibition. The absence of the initiation factor eIF3 (Figure 1, track 17) also prevented the formation of the 48S complex.

The translation initiation on the used m6A-modified mRNAs (both capped and uncapped) demonstrated another type of initiation factors and ATP dependence in standard conditions. As shown previously using capped m^6^A-mRNA, the absence of eIF4F does not result in the complete inhibition of formation of the 48S complex, thus suggesting that the initiation is cap-independent [24]. Our results are consistent with these data. As seen in Figure 1, in the absence of eIF4F, the 48S complex formed, though with lower efficiency, on the capped mRNA (track 8); the direct evidence for the cap-independence of the initiation on the uncapped m^6^A-modified mRNA is shown by the presence (track 3) and absence (track 11) of eIF4F.

The translation of m^6^A-modified mRNA in an ATP-deficient system (Figure 1, tracks 14 and 15) performed in this study is a fundamentally new approach. As shown, the absence of ATP caused no significant inhibition of the 48S complex formation on the modified beta-globin mRNA. An experiment with added eIF4A (R362Q), an inhibitor of ATP-dependent scanning [30], also confirmed the ATP-independent nature of the initiation that occurs on the m^6^A-modified *beta-globin* mRNA.

**Figure 2:**
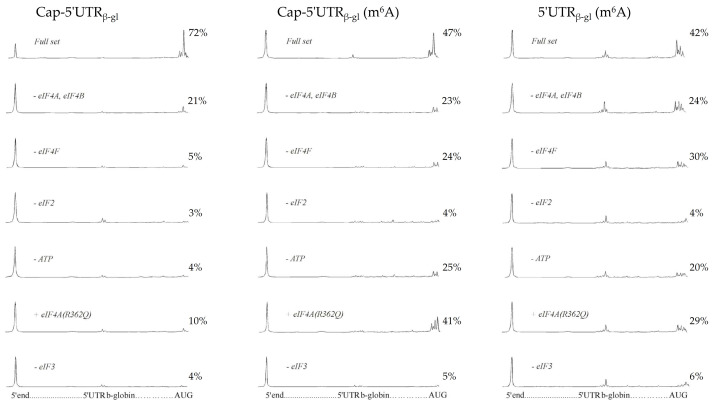
The formation of 48S complexes on mRNAs with the beta-globin leader. Left panel, capped unmodified mRNA (Cap-5′UTR_b-gl_); middle panel, capped m^6^A-modified mRNA (Cap-5′UTR_b-gl_ (m^6^A)); right panel, uncapped m^6^A-modified mRNA (5′UTR_b-gl_ (m^6^A)). Capillary electrophoresis electropherograms of mRNA toeprints are presented. The peak on the 5′ end corresponds to the full-length reverse-transcription product: the peaks on AUG—to 48S complexes. The calculated specific fluorescence intensity is indicated above each 48S peak (%). Quantification is relative (the proportion between peaks) for each electropherogram.

**Figure 3:**
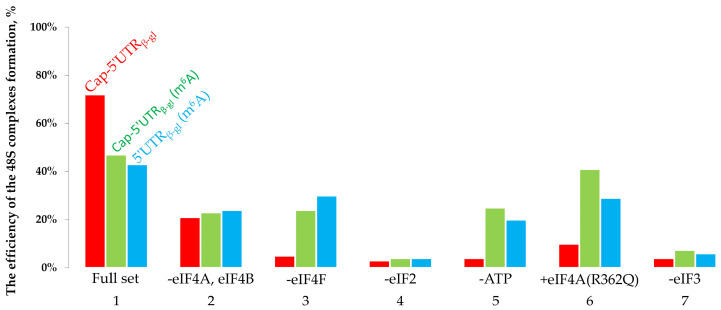
The efficiency of the formation of 48S complexes on mRNAs with the beta-globin leader. Red column, capped unmodified mRNA (Cap-5′UTR_b-gl_); green column, capped m^6^A-modified mRNA (Cap-5′UTR_b-gl_ (m^6^A)); blue column, uncapped m^6^A-modified mRNA (5′UTR_b-gl_ (m^6^A)). The histogram is based on the specific fluorescence intensities corresponding to 48S peaks of the toeprinting reaction (Figure 2). Y-axis, fluorescence percentage at the AUG codon.

To quantify the efficiency of the formation of 48S complexes, capillary electrophoresis was carried out, followed by the detection of fluorescence of toeprinting samples (Figure 2). The specific fluorescence intensity corresponding to each 48S complex peak was calculated [28] (Figure 3).

The specific fluorescence intensities of the 48S peaks demonstrated the efficiency of translation initiation, i.e., the percentage of mRNAs carrying the formed initiation complex. As shown, with the complete set of factors present, 72% of unmodified (control) mRNAs possessed a successfully formed initiation complex. Regarding capped and uncapped m^6^A-modified mRNAs, the percentage was 47% and 42%, respectively (Figure 2 and Figure 3, bar group 1), which indicated that methylation causes a decrease in the initiation along with the elongation decrease [31,32]. The removal of eIF4A and eIF4B from the system (Figure 2 and Figure 3, bar group 2) revealed virtually the same efficiency of initiation on the control mRNA (21%) and on the capped and uncapped m6A-modified mRNAs (23% and 24%, respectively). Notably, the control mRNA showed a much greater decrease in the initiation efficiency than the modified mRNAs (Figure 2 and Figure 3, bar group 1 and 2) compared with the case of the complete set of factors. In other words, the unmodified mRNA is more sensitive to the absence of eIF4A and eIF4B than m^6^A-mRNA.

The removal of eIF4F from the system (Figure 2 and Figure 3, bar group 3) resulted in the virtually complete absence of the initiation complexes from the control mRNA (5%), while for the capped and uncapped m^6^A-modified mRNAs, the initiation efficiency amounted to 24% and 30%, respectively. A similar dependence was observed for the ATP-deficient system: no initiation occurred on the control mRNA, while for the capped m^6^A-modified mRNA, the efficiency was 25%, and 20% for the uncapped one (Figure 2 and Figure 3, bar group 5). The addition of eIF4A (R362Q) entailed a dramatic decrease in the initiation efficiency on the control mRNA (10%) and led to 41% and 29% efficiency on the capped and uncapped m^6^A-modified mRNA, respectively (Figure 2 and Figure 3, bar group 6). In the absence of the initiation factor eIF2 or eIF3, no 48S complex was formed on either of the mRNAs under study (Figure 2 and Figure 3, bar groups 4 and 7).

Thus, methylated mRNAs exhibit lower initiation efficiency than unmodified ones. However, the m^6^A modification allows initiation of mRNA translation in the presence of eIF4A (R362Q), an inhibitor of the ATP-dependent scanning, or in the absence of ATP.

### 2.2. The In Vitro Translation of m^6^A-Modified mRNA

**Figure 4:**
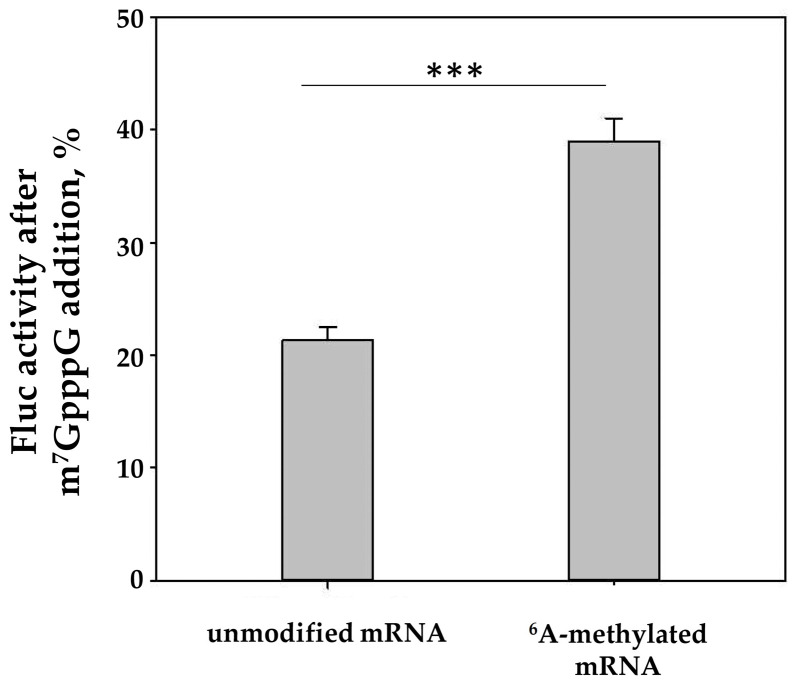
The translation of methylated mRNA shows lower sensitivity to the cap-analog-induced inhibition of cap-dependent translation. C + A + *Firefly luciferase* reporter mRNA carrying the beta-globin 5′ UTR (0.15 nmol) was translated in HEK293T cell extract with or without a cap analog (0.1 mM). The reaction mixture was incubated for 60 min at 30 °C before the determination of Firefly luciferase activity, which was measured as 100% with no cap analog added. The results of three independent experiments are presented. Bars are 2 standard deviations. A two-tailed Student’s t-test was used to estimate the statistical significance; *** *p* < 0.001. The absolute values of luciferase activity are plotted in Supplementary Figure S2.

Next, we checked the extent to which the observed effect of mRNA methylation was pronounced in experiments using a cell-free translation system based on HEK293T cells. For this purpose, the translation of methylated reporter mRNA carrying the rabbit *beta-globin* mRNA 5′-UTR was tested for sensitivity to the addition of a cap analog, a specific inhibitor of cap-dependent translation. Of note, an ATP/m^6^ATP = 1:1 ratio was used for the synthesis of m^6^A-mRNA in the in vitro system. This ratio allowed a high level of m^6^A incorporation into mRNA (Supplementary Figure S1A), while the level of mRNA translation remained sufficiently high (Supplementary Figure S1B). Notably, during the in vitro transcription, incorporation of the modified A’s occurred over the entire mRNA body, including the coding region, thereby, as shown, slowing down the translation elongation at certain codons and affecting the rate of codon-anticodon recognition [31,32]. Figure 4 shows that the methylated mRNA displayed a higher resistance to the inhibition of cap-dependent initiation, which is in good agreement with both the toeprinting results (Figure 1, track 8) and the literature data [24].

**Figure 5:**
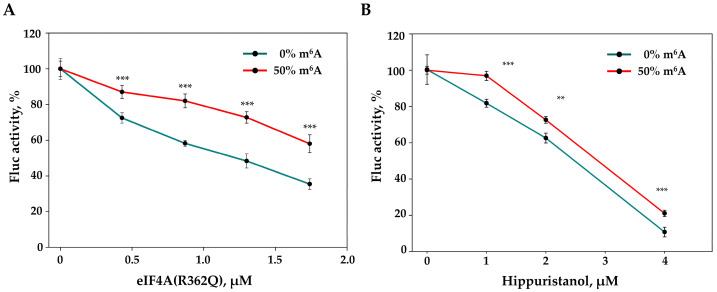
Comparison of translation of unmodified and m^6^A-modified reporter mRNA in the presence of eIF4A (R362Q) (**A**) or hippuristanol (**B**). C + A + *Firefly luciferase* reporter mRNA carrying the beta-globin 5′ UTR (0.15 pmol) was translated in HEK293T cell extract with or without increasing concentrations of recombinant eIF4A (R362Q) (0.43, 0.87, 1.3, and 1.74 µmol) or increasing concentrations of hippuristanol (1, 2, and 4 µmol). The reaction mixture was incubated for 60 min at 30 °C before the determination of Firefly luciferase activity, which was taken to be 100% with no eIF4A (R362Q) or hippuristanol added. The results of three independent experiments are presented. Bars are 2 standard deviations. A two-tailed Student’s t-test was used to estimate the statistical significance; ** *p* < 0.01; *** *p* < 0.001.

Next, we studied the translation of m^6^A-modified and unmodified mRNAs in cell-free systems containing eIF4A (R362Q) and hippuristanol, the inhibitors of ATP-dependent scanning. As seen in Figure 5A, translation of the m^6^A-modified reporter *luciferase* mRNA with the rabbit *beta-globin* mRNA 5′-UTR showed a lower sensitivity to eIF4A (R362Q). However, hippuristanol caused a less-pronounced difference in translation inhibition between the ^6^A-methylated and unmodified mRNAs (Figure 5B).

Due to the elevated affinity of eIF4A (R362Q) for the eIF4F components, this mutant protein removes eIF4F from translation, and the inhibition of eIF4A activity is superimposed on the inhibition of the activity of eIF4F to which the ^6^A-methylated mRNA is less sensitive.

## 3. Discussion

To date, we have no comprehensive explanation of the role of m^6^A in the regulation of protein biosynthesis. So far, it is unclear how exactly this modification affects the initiation of translation on the modified mRNA and the protein biosynthesis in general. We proved that the initiation can occur without recruiting the 5′-end cap-structure. It was reported previously that on modified mRNAs, the translation initiation is independent of the factor eIF4F [24], which was the basis for a conclusion about the cap independence of the initiation on m^6^A-modified mRNA. However, the authors used a cap-containing mRNA; therefore, the conclusion about cap independence was drawn from the function of the factor eIF4F as a cap-binding protein. Here, we present direct evidence for cap independence of the initiation on modified mRNAs. The quantitative analysis of toeprinting products and the evaluation of translation initiation efficiency allowed us to state, for the first time, that although the initiation may occur on uncapped modified mRNA, its efficiency is significantly lower in comparison with unmodified mRNA. Additionally, the considerably lower level of protein synthesis observed on modified mRNAs (Supplementary Figure S1) is indicative of a lower level of elongation efficiency on modified templates. This observation is in agreement with the literature data [31,32].

It should be emphasized that our in vitro experiments used mRNA with m^6^As localized to all feasible positions over the entire mRNA. However, in the cell, mRNA methylation occurs only at specific sites. This suggests that the use of mRNA with a natural methylation profile would allow no additional (nonspecific) effect of methylation on the translation elongation, and probably the initiation as well. Yet, as shown here, even excessive and nonspecific methylation of mRNA confers an advantage over a nonmodified mRNA when the cap-dependent translation is inhibited.

Our study first demonstrates the possibility of ATP-independent translation initiation on m^6^A-modified mRNAs. As shown in this work, ATP hydrolysis energy is not required for the ribosome to reach the AUG codon on modified templates. Hence, a question arises as to the mechanism of translation initiation on modified mRNAs. The authors of the above-cited paper [24] stated that it is scanning that occurs on these mRNAs. This was proved by introducing both a hairpin preventing the ribosome from reaching the AUG codon and an additional upstream AUG codon. In this case, at the downstream AUG codon, the initiation occurred with much lower efficiency or did not occur at all.

We suggest that modified mRNAs have two possible pathways of translation initiation that do not require the energy of ATP hydrolysis. The first is the internal ribosome entry that is close to the AUG codon, probably due to the eIF3-to-m^6^A binding (eIF3 affinity for m^6^A was shown previously [24]). The inhibitory effect of the 5′ UTR hairpin can be explained by the spatial structure of the hairpin, in that it hampers the ribosome binding to mRNA. Alternatively, the ribosome complex can reach the AUG codon on such mRNAs through one-dimensional diffusion (phaseless wandering) along the leader sequence [33,34]. Regardless, we suggest that with m^6^A in the 5′ UTR, the translation initiation pathway is noncanonical. Although the alternative pathway is less efficient, it becomes the only possible one in conditions when no translation initiation occurs on unmodified mRNAs.

So, methylation supports mRNA translation in the case of the suppressed mechanism of cap-dependent translation, which frequently occurs under stress. Whether this plays a role in overcoming stress is currently widely discussed and a focus of current research. For example, mTORC1 (mammalian target of rapamycin complex 1) inhibition, a key event in different types of stress [35], causes inhibition of cap-dependent translation through neutralization of the complex eIF4F. It appears that the suppression of ^6^A methylation in mRNA entails a higher sensitivity of cellular mRNA translation to the inhibition of mTORC1 activity [25]. In other words, in the absence of methylation, mRNA translation displays a higher cap dependence. Interestingly, these data are consistent with those reported by Slobodin et al. [32], who showed that TOP mRNAs, whose translation is most sensitive to mTORC1 activity, carry less numerous m^6^A modifications than other mRNAs because the transcription of TOP mRNAs is more rapid. Under stress, it is TOP mRNA translation that probably must be suppressed first as the most resource-consuming one in the cell, while the translation of the rest of methylated mRNAs remains at its basic level due to the cap-independent mode. Yet, it remains unknown what mechanisms realize the cap-independent translation in this case.

Additionally, it was shown that under stress, the methylation of some mRNAs, whose translation products are required to overcome the stress effects, can increase, thereby provoking a higher level of translation of these mRNAs. For example, HSP70 mRNA is hypermethylated under heat shock, which enables its efficient translation and participation of the synthesized protein HSP70 to remove the heat shock effects [24]. It is suggested that the stress-induced translation of HSP70 mRNA requires the initiation factor eIF3 in immediate interaction with m^6^A and the protein ABCF1, which contributes to TC recruiting.

mRNA methylation can interfere with the translation of some mRNAs. An important pathway that regulates translation under stress is known to be based on eIF2 α-subunit phosphorylation [36]. This modification ultimately reduces the overall level of translation. In contrast, in some mRNAs with several upstream open reading frames (uORFs), the initiation of translation of the main ORF increases as the TC amount decreases. In this case, the ^6^A methylation hampers the initiation of translation of the main ORF since, as shown by [37], it slows down the mRNA scanning, which contributes to a higher probability of translation initiation at the uORF. However, as shown for templates such as ATF4- and GADD45G mRNAs, under stress, they undergo specific demethylation, thereby increasing the probability of translation of the main ORF.

Further investigation is required to clarify how the relative eIF4F/ATP-independence of the translation of modified mRNAs during mRNA scanning promotes mRNA translation both under stress and with suppressed cap-dependent translation.

## 4. Materials and Methods

### 4.1. Plasmids

To synthesize rabbit *beta-globin* mRNA for experiments on the formation of 48S complexes, the plasmid pET28a-BetaGlob-bGlob was used. This plasmid was obtained from pET28 MVHL-STOP [38] by quick change mutagenesis using KOD Hot Start DNA polymerase (Merck Millipore Novagen, Madison, WI, USA) and DNA primers 5′-CGACTCACTATAGGCACTTGCTTTTGACACAACTGTG-3′ and 5′-CACAGTTGTGTCAAAAGCAAGTGCCTATAGT GAGTCG-3′.

For the construction of pSP36TBetaGlobFLucA50, the 5′ UTR of the rabbit beta-globin DNA fragment was obtained by annealing two oligonucleotides (5′-AGCTTACTTGCTTTTGACACAACTGTGTTTACTTGCAATCCCCCAAAACAGACAC-3′ and 5′-CATGGTGTCTGTTTTGGGGGATTGCAAGTAAACACAGTTGTGTCAAAAGCAAGTA-3′; sticky ends similar to those formed after *HindIII* and *NcoI* restriction are shown in bold), followed by their ligation into pSP36TLucA50 [39] and treatment with *NcoI* and *HindIII* restriction endonucleases.

### 4.2. In Vitro Transcription

Rabbit *beta-globin* mRNA was transcribed by T7 RNA polymerase from pET28a-BetaGlob-bGlob linearized with EcoRI. *Firefly luciferase* mRNA with 5′ UTR from rabbit *beta-globin* mRNA was transcribed by SP6 RNA polymerase from pSP36TBetaGlobFLucA50 linearized with *SmaI*. The transcription was performed using a SP6-Scribe Standard RNA IVT Kit (CellScript, WI, USA). For co-transcriptional rabbit *beta-globin* mRNA capping, the GTP concentration in the reaction mixture was reduced to 0.2 mM, and the m^7^G(5′)ppp(5′)G RNA cap structure analog (NEB, Ipswich, MA, USA) was added to 3.8 mM. The capped mRNA transcript for in vitro translation was obtained using a ScriptCap™ m^7^G Capping System and ScriptCap 2′-*O*-Methyltransferase Enzyme (CellScript, Madison, WI, USA) according to the manufacturers’ recommendations.

To obtain m^6^A-containing mRNA for experiments on the formation of 48S complexes, ATP was completely replaced by m^6^ATP at the same concentration (4 mM) in the transcriptional mixture. For translation in a cell-free system, mRNA containing 50% m^6^A was used. To obtain such mRNA, a mixture of 2 mM ATP and 2 mM m^6^ATP was used for in vitro transcription. The quality of the obtained mRNAs was checked by electrophoresis in 6% polyacrylamide gel (PAGE) containing 7 M urea.

### 4.3. Components for Assembling 48S Initiator Complexes

Native factors eIF2, eIF3, and eIF4F, as well as ribosomal 40S subunits, were isolated from rabbit reticulocyte lysate, as described in [40]. Recombinant translation initiation factors eIF1, eIF1A, eIF4A, and eIF4B were obtained according to the protocol presented in the same paper.

### 4.4. Primer Extension Inhibition Assay (Toeprinting)

Formation of the ribosomal initiation complex, and the analysis of the products of the primer extension reaction were performed as described in [27,28,40]. The assembly of the ribosomal 48S initiation complexes was performed from individual purified components of the translation apparatus, namely 40S ribosomal subunits; mRNA; Met-tRNAi, initiation factors eIF1, eIF2, eIF3, eIF4A, eIF4B, and eIF4F; as well as ATP and GMP-PNP (the latter was added to block translation after reaching the initiation codon). The mixture was incubated at 37 °C for 15 min. The primer extension reaction was performed using DNA primer with fluorescent labeling. The cDNAs formed in the primer extension reaction were analyzed by denaturing electrophoresis in 6% polyacrylamide gel. Visualization of cDNA fragments was performed by fluorescence using the Gel Doc System (Bio-Rad, Hercules, CA, USA).

Capillary gel electrophoresis was performed to quantify the ratio of reverse-transcription products. The collected data were processed with GeneMarker 1.5 software (SoftGenetics, State College, PA, USA). Fluorescence intensities corresponding to each cDNA peak were measured to determine the amount of reverse transcription products.

### 4.5. In Vitro Translation

The HEK293T cell extract for a cell-free translation system was obtained as described previously [41].

The translation mixture (10 μL) contained 5 μL HEK293T cell extract, 1 μL 10× translation buffer (200 mM HEPES–KOH, pH 7.6, 10 mM DTT, 5 mM spermidine–HCl, 80 mM creatine phosphate, 10 mM ATP, 2 mM GTP, and 250 μM of each amino acid), 100 mM KOAc, 1 mM Mg(Ac)_2_, 2 units of Human Placental Ribonuclease Inhibitor (Thermo Fisher Scientific, USA), and 0.15 pmol reporter Fluc mRNA. Reaction mixtures were incubated for 45 min at 30 °C, and the luciferase activity was then measured using the OneGlo Luciferase Assay kit (Promega, USA). When indicated, various amounts of m^7^GpppG, ApppG, or hippuristanol, were added, and the translation mix was preincubated for 5 min before the addition of mRNA.

## Supplementary Materials

The following are available online at https://www.mdpi.com/article/10.3390/ijms22073662/s1.
