# Supplementary material for: ATP-Independent Initiation during Cap-Independent Translation of m6A-Modified mRNA
Source: Int J Mol Sci. 2021 Apr 1;22(7):3662. doi: 10.3390/ijms22073662 (PMC8036370; doi:10.3390/ijms22073662)
Supplement: Supplementary file 1 [file ijms-22-03662-s001.pdf]

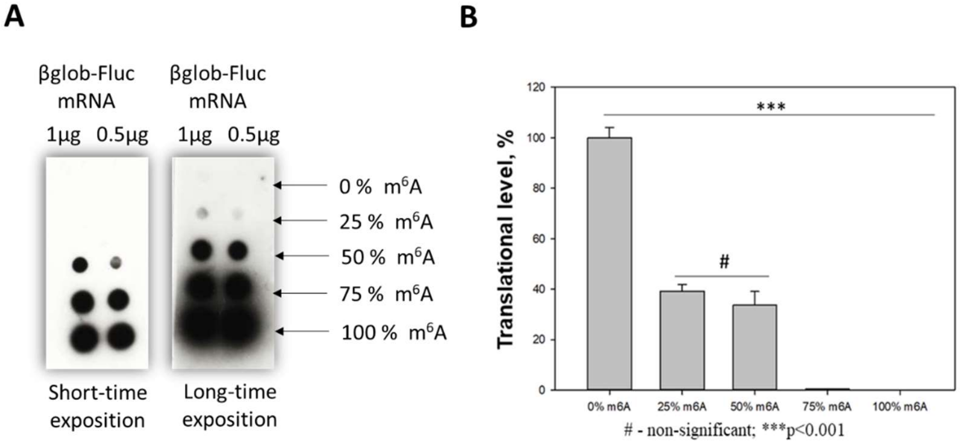

Supplementary figure 1.

In our experiments, we used transcriptionally methylated (m^6A) mRNA where m^6A resided over its entire length, including the coding region. It is known that the presence of adenosine methylated at the N^6 position in the mRNA coding region can significantly reduce the rate of translation elongation of this mRNA (Choi, J .; 2016, Slobodin, 2017). In this regard, it was necessary to find conditions under which FLuc mRNA with 5 'UTR of beta-globin mRNA would have an acceptable level of translation with the greatest amount of m^6A. To obtain the mRNA, an *in vitro* transcription system was used, where the m^6ATP-to-ATP ratio was 0, 25, 50, 75, or 100%. The presence of m^6A in mRNA transcripts was detected using the dot blot analysis **(A)**. For this purpose, mRNA samples were denatured at 70 ° C for 5 minutes. Then an equal volume of chilled 20x SSC buffer was added. Samples were applied onto an Amersham Hybond-N + membrane (GE Healthcare) using the Bio-Dot® Microfiltration System (Bio-Rad). Then, the membrane was treated with UV (1200 μJ/cm2). To remove unbound RNA and block the sites of nonspecific sorption of antibodies, the membrane was incubated for 30-60 min in PBSt buffer (137 mM NaCl, 2.7 mM KCl, 10 mM Na2HPO4, 1.8 mM KH2PO4, 0.1% Tween 20) with 5% non-fat milk added. After that, the membrane was incubated with rabbit antibodies against m^6A (Abcam, USA, # 151230) in the same buffer overnight at 4 ° C. Then, the membrane was washed three times with PBSt, followed by incubation for 1 h in PBSt buffer with 5% non-fat milk and secondary antibodies conjugated with horseradish peroxidase. Immunocomplexes were detected using an ECL Prime kit (GE Healthcare) according to the manufacturer's recommendations.

Next, we checked how the degree of mRNA methylation affects the efficiency of its translation in a cell-free translation system based on HEK293T cell lysate **(B)**. It can be seen that even with a relatively small amount of m^6A in the mRNA transcript, a significant decrease in FLuc activity is observed. Since there is no significant difference between the 50%- and 25%-methylated mRNAs, it was decided to use the former for further experiments.

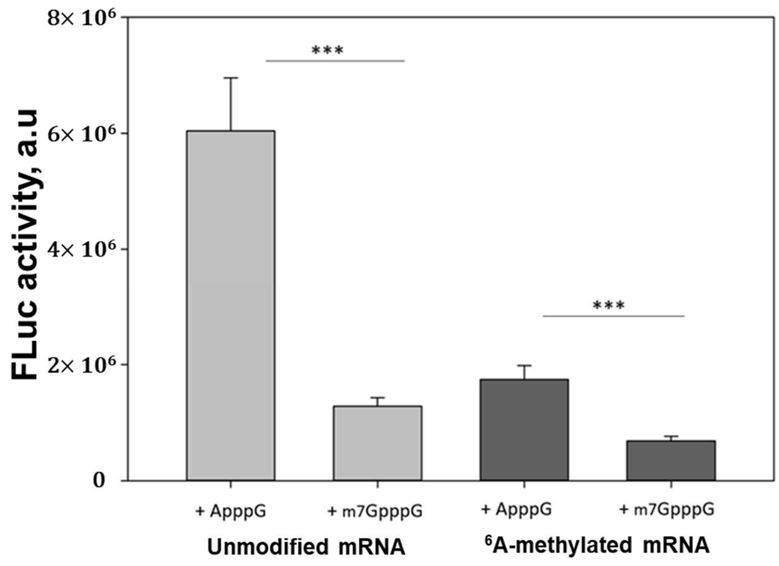

Supplementary figure 2.

The translation of methylated mRNA shows lower sensitivity to the cap-analog-induced inhibition of cap-dependent translation. C + A + Firefly luciferase reporter mRNA carrying the beta-globin 5' UTR (0.15 nmol) was translated in HEK293T cell extract with addition m7GpppG or ApppG(0.1 mM). The reaction mixture was incubated for 60 min at 30°C. The results of three independent experiments are presented. Bars are 3 standard deviations. A two-tailed Student's t-test was used to estimate the statistical significance; ***$p < 0.001$. The relative change in translation after adding a cap-analog is shown in Figure 4.
